# Does a Mobile app improve patients’ knowledge of stroke risk factors and health-related quality of life in patients with stroke? A randomized controlled trial

**DOI:** 10.1186/s12911-019-1000-z

**Published:** 2019-12-21

**Authors:** Yi-No Kang, Hsiu-Nien Shen, Chia-Yun Lin, Glyn Elwyn, Szu-Chi Huang, Tsung-Fu Wu, Wen-Hsuan Hou

**Affiliations:** 1 Institute of Health Policy and Management, College of Public Health, National Taiwan University, Taipei, Taiwan; 2 Evidence-Based Medicine Center, Wan Fang Hospital, Taipei Medical University, Taipie, Taiwan; 3 Department of Intensive Care Medicine, Chi Mei Medical Center, Tainan, Taiwan; 4 Department of Physical Medicine and Rehabilitation, Taipei Medical University Hospital, Taipei, Taiwan; 5 The Dartmouth Institute for Health Policy and Clinical Practice, Dartmouth College, New Hampshire, USA; 6 Scientific Institute for Quality of Healthcare, University Nijmegen Medical Centre, Armsterdam, Netherlands; 7 Cochrane Institute for Primary Care and Public Health, Cardiff University, Cardiff, UK; 8 Master Program in Long-Term Care and School of Gerontology Health Management, College of Nursing, Taipei Medical University, No. 250, Wu-Hsing Street, Xinyi District, Taipei City, 11031 Taiwan; 9 Center of Evidence-Based Medicine, Taipei Medical University Hospital, Taipei, Taiwan

**Keywords:** Stroke, Stroke knowledge, Health-related quality of life, Health-education app, Mobile health care

## Abstract

**Background:**

Developing a stroke health-education mobile app (SHEMA) and examining its effectiveness on improvement of knowledge of stroke risk factors and health-related quality of life (HRQOL) in patients with stroke.

**Methods:**

We recruited 76 stroke patients and randomly assigned them to either the SHEMA intervention (*n* = 38) or usual care where a stroke health-education booklet was provided (n = 38). Knowledge of stroke risk factors and HRQOL were assessed using the stroke-knowledge questionnaire and European Quality of Life–Five Dimensions (EQ-5D) questionnaire, respectively.

**Results:**

Sixty-three patients completed a post-test survey (the SHEMA intervention, *n* = 30; traditional stroke health-education, *n* = 33). Our trial found that patients’ mean knowledge score of stroke risk factors was improved after the SHEMA intervention (*Mean difference* = 2.83; *t* = 3.44; *p* = .002), and patients’ knowledge was also improved in the after traditional stroke health-education (*Mean difference* = 2.79; *t* = 3.68; *p* = .001). However, patients after the SHEMA intervention did not have significantly higher changes of the stroke knowledge or HRQOL than those after traditional stroke health-education.

**Conclusions:**

Both the SHEMA intervention and traditional stroke health-education can improve patients’ knowledge of stroke risk factors, but the SHEMA was not superior to traditional stroke health-education.

**Trial registration:**

NCT02591511 Verification Date 2015-10-01.

## Highlights

- Health-education mobile app has no better effect to improve knowledge of stroke risk factors compared to health-education booklet.
- Health-education mobile app may borderline improve health-related quality of life compared to health-education booklet.
- Younger patients (aged ≤55 years) might have greater improvement in knowledge of stroke risk factors.

## Background

Stroke, a cerebrovascular disease, is one of the top three global causes of death and long-term disability globally [1]. To manage chronic diseases and long-term disabilities requires patients’ active participating in self-management [2] and preventing complications or recurrence after discharge [3]. In Taiwan, there are approximately one fourth stroke patients readmitted within three months of discharge due to recurrent stroke in Taiwan [4]. A 17-years follow-up study reported that death rate among patients with recurrent strokes were higher than those with the first-time strokes [5], along with severe disabilities [6]. Therefore, healthcare providers should provide patients with stroke both accurate information regarding how to prevent stroke recurrence and living assistance to reduce stroke care burden [7].

Health-education about stroke is a tool for cultivating attitudes and skills to reduce the risks of stroke-induced disability and mortality. Unfortunately, this kind of education, an essential part of stroke care, has not yet been completely integrated in regular clinical settings [8]. Moreover, few stroke-education materials comprehensively cover relevant information regarding the risk factors of recurrent stroke in various conditions [9]. Health-education mobile app is an approach to reach patients in today, and this kind app is distinguished by their use of text, animations, or pictures to share health information regarding user care or education. As a result of technological advancement, many health-related services are now assisted by information and communications technology, which provide patients direct and immediate access to health-related messages through their smartphones. That is to say, healthcare has a way to improve speed and reduce manpower through advanced technology [10, 11]. Therefore, mobile healthcare not only become an effective approach for health-education, but also improve accessibility to medical information [11].

World Stroke Organization developed the Stroke Riskometer app for healthy individuals to easily calculate their stroke risk indexes [12]. However, the content of the app is limited to primary stroke prevention and does not include risk factors related to secondary prevention. Several studies reported that improvements of health-related knowledge were significantly associated with use of mobile healthcare apps [13–16]. Nevertheless, none of these apps focused on health-education for risk factors related to the secondary stroke prevention.

App is an approach to deliver health information to patients and provide patients another form in getting health education. Patients can empower themselves through traditional materials or app according to their preference. This study therefore developed a mobile healthcare app, stroke health-education mobile app (SHEMA), for the comparison of stroke health-education booklet in improving patients’ knowledge of stroke risk factors. The purpose of this study aimed to examine the effectiveness of the SHEMA in improving patients’ knowledge of stroke risk factors and health-related quality of life (HRQOL) in patients with stroke in Taiwan. Our hypotheses were: (1) patients’ knowledge of stroke risk factors and HRQOL after the SHEMA intervention were better than before the SHEMA intervention; (2) patients in the SHEMA intervention group had better knowledge of stroke risk factors and HRQOL than patients traditional stroke health-education group after experimental period.

## Methods

This was a single blind (assessor) randomized controlled trial (RCT). Block randomization was performed on the basis of 4 units. Random numbers were calculated using computer software. Serial numbers were placed in opaque sealed envelopes. A researcher was responsible for recruitment, defining measures, pretest composition, randomization, and group assignment. Two research assistants who were blinded to randomization and allocation collected post-test data. Both of them participated in the completion of the prearrangement process before a case was closed. They were also prohibited to contact with patient because any interaction with patients may bias study outcome. The study protocol was approved by the Taipei Medical University Joint Institutional Review Board, and the study was registered in the ClinicalTrials.gov (NCT02591511).

### Participants

We estimated our sample size using G-Power (version 3.1; Franz Faul. Christian-Albrechts-Universität, Kiel, Germany). The study was designed to have a power of 80% to detect the intervention group compared with the control group, assuming a two-sided alpha level of 0.05. The intervention and control groups were assigned using a 1:1 ratio and considered 15% dropout rate in both groups. As a result, 64 patients were the minimum requirement for this study. We recruited patients with stroke from the stroke wards in four teaching hospitals in Taiwan between September 2014 and April 2016. The following inclusion criteria were used: (a) stroke diagnosis by medical doctors (international classification of disease, 9th edition (ICD-9): 430–438), (b) history of the first stroke onset, (c) ability to communicate in daily life without difficulty and clearly explain the meaning of reading material verbally, (d) no serious cognitive deficits (Mini–Mental State Examination score ≥ 24), (e) use of a smartphone with experience using apps, and (f) agree to participate in the study and follow the instructions. The exclusion criteria were as follows: (a) involvement in health education–related work or being a medical staff member (both of which could affect the results) and (b) failure to follow the instructions of the tester or presence of conditions (eg, severe aphasia or delirium) that may affect responses. We clearly explained all the procedures in this trial, and patients completed informed consent before data collection.

### Intervention

After randomization, we provided a stroke health-education booklet and a stroke health-education mobile app (SHEMA) with same stroke-related health information (https://play.google.com/store/apps/details?id=com.soohoobook.healtheducation&fbclid=IwAR1ctyMkwBzw3el5IA2O3aYaqHyVF9dm8R6f4zcFzRtRGI_QyAleXl6d2YE) for control group and intervention group respectively. We asked the patients to read the booklet or the SHEMA content at home for 7–14 days, and five minutes per day was minimal requirement.

About the control group, the stroke health-education booklet mainly contained information regarding stroke risk prevention. The stroke health-education content covered 12 topics of risk factors in stroke patients such as: stroke history, heart disease, age, irregular work and sleep patterns, obesity, family history and genetic factors, hyperlipidemia, hypertension, unbalanced diet, diabetes mellitus, changes in ambient temperature, and sex. A trained research assistant provided standard poststroke health-education as follows:

1. Provided stroke-related health-education booklets to patients.
2. Used about 45 min to explain the booklet content to the patients.

On the other hand, patients in the intervention group received the SHEMA. It could be downloaded for free and allowed patients to select their own educational content. The content covered the same 12 health-education topics as the aforementioned booklet. The patients can use the SHEMA according to their time and needs, and the app was completely open for the patients. Thus, they can browse the content for several time without time and location limitation. Another trained research assistant provided the SHEMA as follows:

1. Assisted the patients in installing the SHEMA onto their personal smartphones.
2. Used about 45 min to explain the SHEMA content and its method of operation to the patients.

### Measures

We asked the patients to complete post-intervention questionnaires within 30 days after completion of the intervention. A research assistant was responsible for collecting the completion of patients’ knowledge of stroke risk factors and European Quality of Life–Five Dimensions (EQ-5D) questionnaires.

Stroke-knowledge questionnaire was our primary outcome, which assess patients’ knowledge of stroke risk factors from the change score of stroke-knowledge questionnaire before and after the intervention. The questionnaire comprised 3-item equivalent single-choice questions edited from the content of educational materials. The question content was based on the stroke health-education booklet and adapted to app format for this study. Knowledge regarding each of the 12 risk factors was assessed by using three questions. Each question was one point, and total score was 36 (3 × 12 = 36). The higher the score, the better the outcome was. If posttest score was higher than the pretest score, it indicated that patients’ knowledge was improved.

EQ-5D, a reliable tool for assessing HRQOL, were the secondary outcome in our study, and it involves two parts including the EQ-5D index and visual analog scale (VAS). In the index section, patients are asked to answer five questions regarding their mobility, self-care, daily activities, pain and discomfort, and anxiety and depression. Each question has three possible responses: no, moderate, and extreme (one, two, and three points, respectively). The total score ranges from five to 15 points. A health survey in Taiwan indicated that the EQ-5D is an utility-based assessment of HRQOL [17]. Our study used the EQ-5D index in Taiwanese version. Higher index score indicates higher HRQOL [18]. About the VAS section, patients were required to indicate their current state of health using a score range of 0 to 100. Lower value represents poorer health conditions, and 100 refers to the optimal health condition. The EQ-5D has high test–retest reliability and validity [17].

### Statistical analysis

According to our research purpose, we selected analysis methods. The results were analyzed using per-protocol analysis. We followed common threshold (*P* < 0.05) when we judged statistical significance. In addition, we calculated Cohen’s d for determining effect size. Usually, a Cohen’s d of > 0.8, > 0.5, and < 0.2 indicates large effect, moderate effect, and poor effect, respectively [19]. SPSS (version 18.0; SPSS Inc., Chicago, IL, USA) was used for data analysis.

Number, percentage, mean, and standard deviation were used to describe age, sex, educational level, stroke type, and disability assessment scores in both the groups. Using the chi-square test, we analyzed sex, education level, stroke type, and disability to determine whether significant differences existed between the intervention and control groups. Moreover, chi-square tests were used for subgroup analyses to detect the difference of patient numbers improvements in stroke knowledge. Using the independent sample *t* test, we determined between-group differences in the age and preintervention and postintervention scores of stroke knowledge and HRQOL. Using the paired *t* test, the patients’ knowledge of stroke risk factors and HRQOL before and after the intervention were compared in both groups.

## Results

**Fig. 1:**
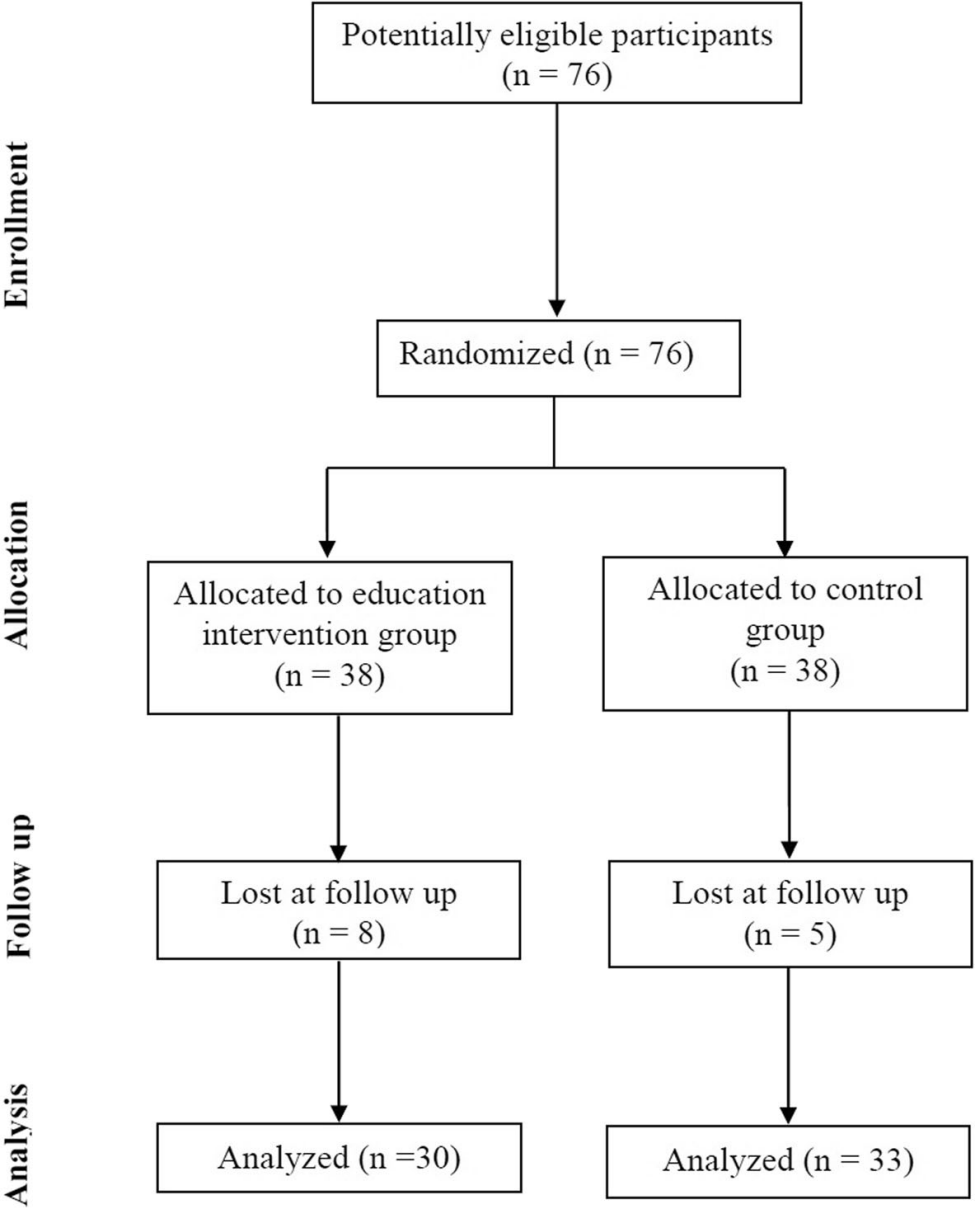
Flow of patient enrollment and study completion according to the consolidated standards of reported trial statements

We randomly and equally assigned 76 patients with stroke to the intervention and control groups (*n* = 38 in each). Of them, 13 patients (8 and 5 from the intervention and control groups, respectively) were lost to follow-up; the main reasons were as follows: cease of communication (*n* = 6), failure to return for follow-up within the scheduled time (n = 6), and rejection of posttest (*n* = 1). Finally, 63 participants completed all test procedures; of them, 30 and 33 were in the intervention and control groups, respectively (Fig. 1). No significant intergroup differences in the demographics, stroke types, or modified Rankin scale (mRS) scores were observed (all *P* > 0.05; Table 1). The mRS is used to measure the degree of disability in patients with stroke. Because no patient exhibited complete absence of symptoms (Level 0), severe disability (Level 5), or death (Level 6), these levels are not listed in Table 1.

**Table 1:** Patient characteristics (*n* = 63)

Characteristics	Intervention (*n* = 30)	Control (n = 33)	*Χ*^*2*^/*t*	*P*
Demographic				
Age	50.47 ± 10.82	52.33 ± 11.03		0.501
Sex			0.081	0.777
Male	21 (70.0)	22 (66.7)		
Female	9 (30.0)	11 (33.3)		
Educational background			0.616	0.735
Junior high school or lower	4 (13.3)	4 (12.1)		
High school	9 (30.0)	13 (39.4)		
University or above	17 (56.7)	16 (48.5)		
Stroke severity				
Type			0.896	0.446
Infarction	11 (36.7)	16 (48.5)		
Hemorrhage	19 (63.3)	17 (51.5)		
Modified Rankin scale level			2.264	0.519
1	6 (20.0)	9 (27.3)		
2	13 (43.3)	10 (30.3)		
3	7 (23.3)	6 (18.2)		
4	4 (13.3)	8 (24.2)		
Stroke-knowledge questionnaire				
Total score	26.23 ± 6.65	25.21 ± 5.54		0.509
Health-related quality of life (EQ-5D)				
Index	0.55 ± 0.29	0.54 ± 0.29		0.882
Visual analog scale	59.67 ± 20.17	61.36 ± 22.34		0.754

Data were mean ± SD or n (%)

No significant differences between the intervention and control groups were discovered for stroke knowledge (*P* = 0.51), EQ-5D index (*P* = 0.88), or EQ-5D VAS (*P* = 0.75) before the intervention. As for the intervention group, the overall total stroke-knowledge questionnaire scores were significantly higher in the posttest (29.07 ± 5.27) than in the pretest (26.23 ± 6.65; *P* = 0.002), while the EQ-5D index (0.62 ± 0.29 vs 0.55 ± 0.29, *P* = 0.11) and EQ-5D VAS score (62.30 ± 18.77 vs 59.67 ± 20.17, *P* = 0.45) were both higher than those in the pretest, but the differences were nonsignificant (Table 2). However, the mean change score of stroke-knowledge questionnaire, EQ-5D indexes, and EQ-5D VAS scores were respectively 28.00 ± 5.46 and 29.04 ± 5.27 (Cohen’s d = 0.194; *P* = 0.43), 0.46 ± 0.41 and 0.62 ± 0.29 (Cohen’s d = 0.451; *P* = 0.07), and 65.00 ± 18.37 and 62.30 ± 18.77 (Cohen’s d = − 0.145; *P* = 0.57; Table 3).

**Table 2:** Comparison of pretest and posttest scores in intervention and control groups

Outcome	Pretest	Posttest	Mean difference	*t*	95% CI	*P*
Stroke-knowledge questionnaire						
Total score						
Intervention ^a^	26.23 ± 6.65	29.07 ± 5.27	−2.83 ± 4.51	−3.440	−4.52 to −1.15	0.002*
Control ^b^	25.21 ± 5.54	28.00 ± 5.46	− 2.79 ± 4.36	−3.676	− 4.33 to − 1.24	0.001**
Health-related quality of life (European Quality of Life–Five Dimensions)						
Index						
Intervention ^a^	0.55 ± 0.29	0.62 ± 0.29	−0.07 ± 0.22	−1.640	−0.15 to 0.02	0.11
Control ^b^	0.54 ± 0.40	0.46 ± 0.40	0.08 ± 0.34	1.426	−0.04 to 0.20	0.16
Visual analog scale						
Intervention ^a^	59.67 ± 20.17	62.30 ± 18.77	−2.63 ± 18.81	−0.767	−9.66 to 4.39	0.45
Control ^b^	61.63 ± 22.33	65.00 ± 18.37	−3.64 ± 19.89	−1.050	−10.69 to 3.41	0.30

*P < .01; **P < .001. ^a^ n = 30; ^b^ n = 33; CI, confidence interval; data were mean ± SD

**Table 3:** Comparison of intervention and control groups

Outcome	Intervention ^a^	Control ^b^	Cohen’s d	*t*	95% CI	*P*
Stroke-knowledge questionnaire						
Total score	29.07 ± 5.27	28.00 ± 5.46	0.194	0.788	−1.64 to 3.78	0.43
Health-related quality of life (European Quality of Life–Five Dimensions)						
Index	0.62 ± 0.29	0.46 ± 0.41	0.451	1.825	−0.01 to 0.34	0.07
Visual analog scale	62.30 ± 18.77	65.00 ± 18.37	−0.145	−0.577	−12.06 to 6.66	0.57

^a^ n = 30; ^b^ n = 33; CI, confidence interval; data were mean ± SD

**Fig. 2:**
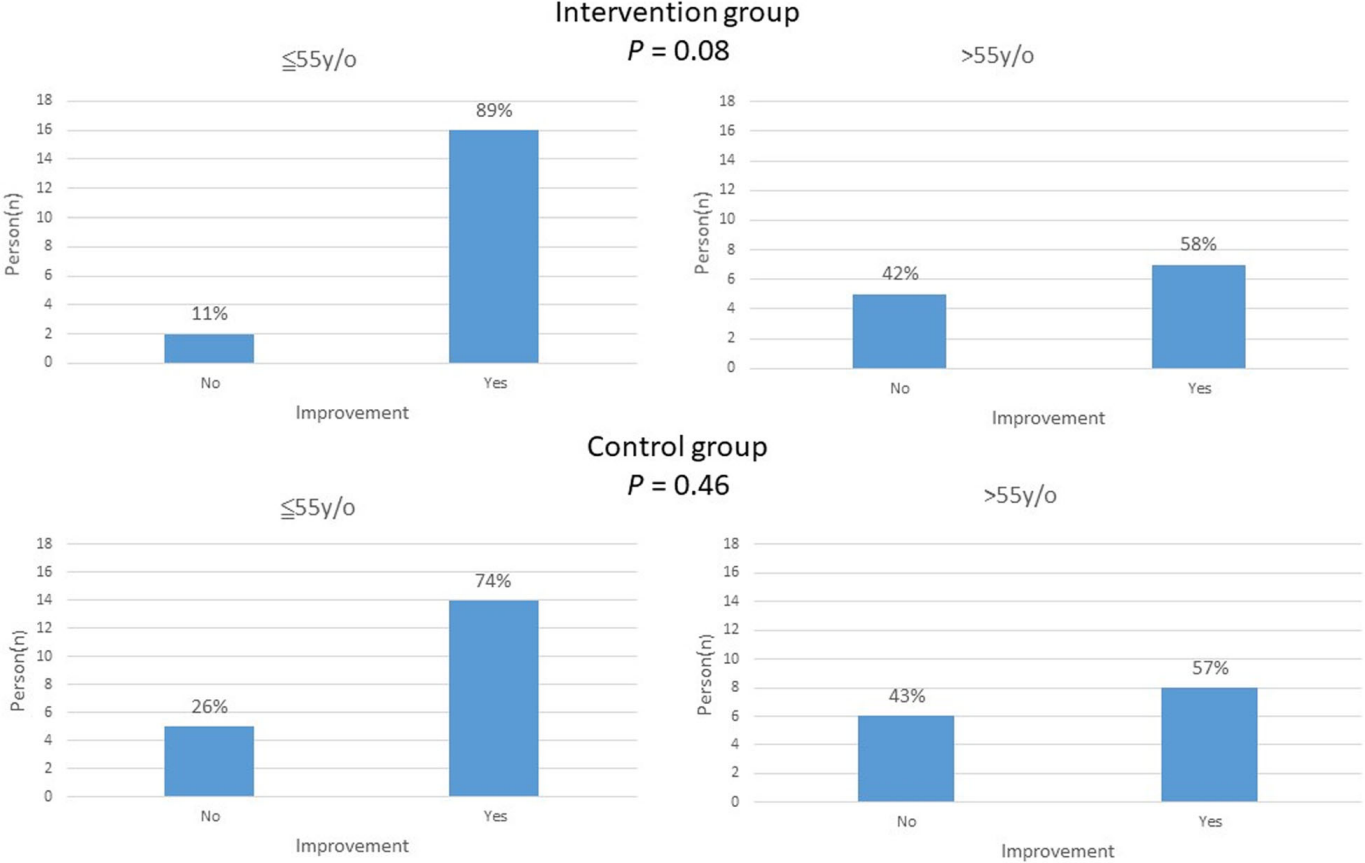
Comparison of knowledge improvement percentage among different age subgroup

Subgroup analysis compared numbers/percentages of stroke-knowledge improvement in patients aged > 55 and ≤ 55 years. The results revealed that 89 and 58% patients’ stroke-knowledge improvements in the intervention group (*P* = 0.08; Fig. 2) while 74 and 57% patients’ stroke-knowledge improvements in the control group (*P* = 0.46; Fig. 2), respectively. The subgroup analysis for EQ-5D index outcome revealed nonsignificant results (*P* = 0.14 and 0.48 for the intervention and control groups, respectively). Nonsignificant results were also in the subgroup analysis for EQ-5D VAS (*P* = 0.71 and 0.49 for the intervention and control groups, respectively).

## Discussion

In overall, patients’ knowledge of stroke risk factors was improved in both the SHEMA and control groups after our health-education although we did not find significant difference in stroke knowledge between the intervention and control groups. On the other hand, no significant pretest–posttest difference in HRQOL were noted in neither the intervention nor the control group. However, compared to the the traditional health-education booklet, SHEMA intervention might be more effective in improving EQ-5D index and younger patients’ (aged ≤55 years) stroke risk factor knowledge because there was a marginal significance for difference in EQ-5D index and improvement patient numbers between the two groups.

### Effects on patients’ knowledge of stroke risk factors

Previous RCTs have reported significant improvements in health-related knowledge after interventions with mobile healthcare apps [13–16]. However, in this study, no significant difference was noted between the intervention and control groups. One potential reason for this non-significant finding may be homogeneous population with high education in both groups. Because about half cases in each group graduated from university or graduate school, their learning capacity may be a strong factor affecting our outcomes, especially in knowledge learning. The other reason may be monitoring of intervention. A previous study used a longer intervention period and an app that combined in-person education sessions with remote daily monitoring of medication compliance using “selfie videos”. Their results indicated that the disease-related knowledge of young people with sickle cell disease increased after a 90-day intervention [13]. However, our app-based intervention only lasted for approximately 30 days and lacked remote daily monitoring. This may have hampered stroke-knowledge improvement. As comparing with standard care, the use of a combination of self-care protocols and a simple atrial fibrillation–related app containing clinical decision-making tools, educational materials, and patient involvement strategies significantly improved the relevant knowledge of patients with atrial fibrillation [14]. However, our patient involvement strategy was relatively weak, which may have led to the absence of significant differences between the outcomes for the intervention and control groups.

Some apps include visual reminders or animations to enhance the effects of an intervention [15, 16]. However, in the current intervention, we provided the patients with only the information regarding the content of the SHEMA at the beginning of the intervention. Rather than automatically sending text messages, we asked patients to read the content at home, once a day, for at least 5 min. Moreover, no pictures or animations were included to attract the patients to use the SHEMA. Therefore, more delicate design or advanced layout is necessary to facilitate the effectiveness of SHEMA.

The use of mobile health apps could significantly improve patients’ clinical communication and satisfaction in Chinese outpatient public hospitals [20]. In addition, rapidly accessible and comprehensive information can provide easy access to health information compared with traditional bulky reference books [21]. This RCT study resolved the aforementioned Chinese study’s limitations of selection bias and absence of comparison cohort; however, our results remained nonsignificant, potentially because of the insufficient sample or effect size used. A recent population-based survey reported that smartphone users were younger (average age, 47.9 years old), performed more health information research, and were more health literate [22]; this may explain our result that younger patients achieved relatively better improvement in patients’ knowledge of stroke risk factors. Thus, in the future, specific implementation of the health-education app to patients from younger generations may generate desirable results. Further research by using such apps for more participants over longer periods with clearer pictures and animations must be conducted.

### Effect on HRQOL

Our study observed that the SHEMA does not lead to better HRQOL. In fact, previous studies about effects of health management apps on HRQOL are controversial [23–25]. The inconsistency among studies may be due to different diseases and app designs. As we know that stroke, diabetes, and asthma are different diseases, and therefore, health education apps intervention may effect on HRQOL those patients differently. For instance, patients with asthma can avoid breathing difficulty or release the symptom through changing their lifestyle. Then, their HRQOL may be improved when they acquire relevant information or knowledge from health management app [25]. In contrast, patients after stroke are usually challenged by irreversible disability. Patients’ HRQOL might be slightly improved through acquiring more relevant knowledge, applying in stroke self-management, and promoting healthier behavior after stroke. Hence, health education app has limited benefits of HRQOL on patients with stroke. Moreover, our app only provided health-education content without an interactive platform to provide and reply patients’ information and questions. To determine the obstacles affecting HRQOL improvement after using educational apps, research must focus on progressive innovation, change, and identification of service functions that effectively improve patient HRQOL.

### Study strengths

First, randomized group allocation with block stratification eliminated potential confounding factors and produced even sample sizes for the two groups, thus preventing any selection bias. Second, although 17% of the patients lost to follow-up is acceptable, sensitivity analysis was performed and revealed that no significant differences between the baseline characteristics of the respondents and non-respondents.

### Limitations

First, most of our participants are young and well educated recruited from urbanized cities, who already obtained high averaged knowledge scores in both groups’ pre-tests (72 vs 69% correction rate). This might have had an influence on the result and limit the generalizability. Second, the sample size is small (i.e., a total of 63 patients recruited) and the intervention period was less than 1 month, which may have been insufficient for further examining the age interfering with intervention effects on the two educational designs of stroke risk education, or improving patients’ knowledge and HRQOL. In the future, a longer intervention period with interaction analyses is required to examine the effects of the SHEMA in patients with stroke. Third, our app only conveyed stroke risk factors-related information through plain text format. The addition of pictures, animations, and patient involvement strategies would benefit future educational app designs.

### Practice implications

Educating stroke patients about the risk factors to prevent stroke recurrence is crucial for effective stroke care. Although our study resulted that the SHEMA had no significant effect on knowledge; however, younger patients (aged ≤55 years) might prone to demonstrate better stroke-knowledge improvement. The American Heart Association and American Stroke Association provided evidence-based recommendations for assessing risk factors online to prevent recurrent stroke [26]; accordingly, this app intervention integrated timely information with easy accessibility for stroke patients. Therefore, clinical professionals may incorporate mobile apps into their standard health care procedures to improve the health of patients with stroke.

## Conclusion

Although no significant effect on improving patients’ knowledge of stroke risk factors after a short-term intervention, the SHEMA group shows borderline improvement EQ-5D index compared to the control group. In addition, larger numbers of younger patients (aged ≤55 years) tend to improve their stroke-knowledge knowledge in SHEMA group than the control group. Because the SHEMA provides timely information with easy accessibility for stroke patients, it may worth to be applied to clinical practice. Nevertheless, we still anticipate further study to investigate the effectiveness of the app in secondary stroke prevention.

## Data Availability

All data generated or analyzed during this study are included in this published article.

## Abbreviations

App: application
EQ-5D: European Quality of Life–Five Dimensions
HRQOL: health-related quality of life
RCT: randomized controlled trials
VAS: visual analog scale
